# Omega-3 Polyunsaturated Fatty Acids And Adipose Tissue Inflammation: Longitudinal Analysis in the PROMISE Cohort

**DOI:** 10.1210/clinem/dgae445

**Published:** 2024-06-29

**Authors:** Ji-Eun Chon, Zhila Semnani-Azad, Kira Zhi Hua Lai, Phillip W Connelly, Ravi Retnakaran, Stewart B Harris, Adam H Metherel, David J A Jenkins, Richard P Bazinet, Anthony J Hanley

**Affiliations:** Department of Nutritional Sciences, Temerty Faculty of Medicine, University of Toronto, Toronto, ON M5S 1A8, Canada; Department of Nutritional Sciences, Temerty Faculty of Medicine, University of Toronto, Toronto, ON M5S 1A8, Canada; Department of Nutrition, Harvard T.H. Chan School of Public Health, Boston, MA 02115, USA; Department of Nutritional Sciences, Temerty Faculty of Medicine, University of Toronto, Toronto, ON M5S 1A8, Canada; Keenan Research Centre for Biomedical Science, St. Michael's Hospital, Toronto, ON M5B 1M4, Canada; Division of Endocrinology and Metabolism, University of Toronto, Toronto, ON M5S 3H2, Canada; Department of Laboratory Medicine and Pathobiology, University of Toronto, Toronto, ON M5S 1A8, Canada; Division of Endocrinology and Metabolism, University of Toronto, Toronto, ON M5S 3H2, Canada; Lunenfeld-Tanenbaum Research Institute, Mount Sinai Hospital, Toronto, ON M5G 1X5, Canada; Leadership Sinai Centre for Diabetes, Mount Sinai Hospital, Toronto, ON M5G 1X5, Canada; Department of Family Medicine, Western University, London, ON N6G 2M1, Canada; Department of Nutritional Sciences, Temerty Faculty of Medicine, University of Toronto, Toronto, ON M5S 1A8, Canada; Department of Nutritional Sciences, Temerty Faculty of Medicine, University of Toronto, Toronto, ON M5S 1A8, Canada; Toronto 3D Knowledge Synthesis and Clinical Trials Unit, Clinical Nutrition and Risk Factor Modification Center, St. Michael's Hospital, Toronto, ON M5C 2T2, Canada; Clinical Nutrition and Risk Factor Modification Centre, St. Michael's Hospital, Toronto, ON M5B 1W8, Canada; Department of Medicine, Temerty Faculty of Medicine, University of Toronto, Toronto, ON M5S 1A8, Canada; Li Ka Shing Knowledge Institute, St. Michael's Hospital, Toronto, ON M5B 1W8, Canada; Division of Endocrinology and Metabolism, St. Michael's Hospital, Toronto, ON M5B 1W8, Canada; Department of Nutritional Sciences, Temerty Faculty of Medicine, University of Toronto, Toronto, ON M5S 1A8, Canada; Department of Nutritional Sciences, Temerty Faculty of Medicine, University of Toronto, Toronto, ON M5S 1A8, Canada; Division of Endocrinology and Metabolism, University of Toronto, Toronto, ON M5S 3H2, Canada; Leadership Sinai Centre for Diabetes, Mount Sinai Hospital, Toronto, ON M5G 1X5, Canada; Dalla Lana School of Public Health, University of Toronto, Toronto, ON M5T 3M7, Canada

**Keywords:** Omega-3 polyunsaturated fatty acids, adipose tissue inflammation, observational, obesity

## Abstract

**Objectives:**

Although preclinical studies have shown a beneficial impact of omega-3 (n-3) polyunsaturated fatty acids (PUFAs) on adipose tissue (AT) inflammation, the current literature from human studies is limited. Therefore, we aimed to evaluate the longitudinal associations of circulating levels of n-3 PUFAs with biomarkers of AT inflammation.

**Methods:**

Longitudinal data from participants in the PROMISE cohort (n = 474) were used. AT inflammation was measured using circulating biomarkers at baseline and up to 2 follow-up visits. n-3 PUFAs were measured at baseline in 4 serum lipid fractions. Generalized estimating equations analyses evaluated longitudinal associations between n-3 PUFAs and AT inflammation, adjusting for covariates.

**Results:**

Fully adjusted generalized estimating equation models indicated that higher baseline proportions of eicosapentaenoic acid, n-3 docosapentaenoic acid, and docosahexaenoic acid in total serum were significantly inversely associated with longitudinal change in soluble CD163 (all *P* < .05). A significant positive association of n-3 docosapentaenoic acid and docosahexaenoic acid with longitudinal change in adiponectin (*P* < .05) was also observed. Generally consistent associations were observed between n-3 PUFAs and soluble CD163 and adiponectin in the four lipid fractions.

**Conclusion:**

These findings will add to the limited evidence on the potential role n-3 PUFAs have in the prevention and management of AT inflammation in humans and may help inform future interventions targeting chronic inflammation at the level of AT.

Currently, approximately one third of the world's population is living with obesity, making it one of the most significant global population health issues (1). The worldwide prevalence of obesity is predicted to increase from ∼30% to 35% over the next 10 years (2), intensifying its clinical, public health, and economic impact arising from the associated short- and long-term sequelae (3-5).

The mechanisms underlying obesity's link with chronic disease etiology are complex and multifactorial, although the role of adipose tissue (AT) as the primary origin of inflammation has become a topic of increasing interest given that chronic low-grade inflammation is a central player in a range of obesity-associated chronic diseases (5, 6). The development of obesity is characterized by hypertrophy and hyperplasia of adipocytes, resulting in the recruitment of M1 pro-inflammatory macrophages (6-8). The activation of this M1 macrophage pathway results in a shift toward a pro-inflammatory AT phenotype and the secretion of pro-inflammatory cytokines including TNF-α from the adipocytes (6, 9, 10). As a result of this accumulating evidence, inflammation in AT is considered a promising target for early intervention for inflammation-driven chronic diseases, and thus identifying strategies to dampen inflammation at the level of AT has become a scientific priority (6, 8, 11-13). Drug candidates such as thiazolidinediones and IL-1β antagonists have produced mixed results in ameliorating AT inflammation, and some have notable side effects (6). Therefore, there is an urgent need for research focusing on nonpharmacological approaches to modulate inflammation in AT.

Omega-3 (n-3) polyunsaturated fatty acids (PUFAs) are not produced de novo in humans; alpha-linolenic acid (ALA) (an essential nutrient) (11, 14) is obtained from the consumption of flaxseed, canola, and soybean, whereas eicosapentaenoic acid (EPA), n-3 docosapentaenoic acid (n-3 DPA), and docosahexaenoic acid (DHA), can be obtained from marine animal sources such as salmon (15). Studies in animal models have documented a beneficial impact of n-3 PUFAs on AT inflammation through multiple pathways (6, 13, 16-18). For example, in a mouse model, it was found that fish oil supplementation exerted an anti-inflammatory effect on the cross-talk between CD8^+^ T cells and adipocytes by increasing mRNA expression of anti-inflammatory proteins and increasing the chemotaxis of anti-inflammatory macrophages (19). However, there have been only a limited number of smaller short-term randomized controlled trials (RCTs) and no longitudinal observational cohort studies that we are aware of examining the effects of n-3 PUFAs on AT inflammation in healthy humans (20-23). Further, associations of n-3 PUFAs in specific lipid fractions (cholesteryl ester [CE], phospholipid [PL], triacylglycerol [TG], and nonesterified fatty acids [NE]) have not been examined; this issue is potentially important given their unique nutritional and metabolic characteristics.

Our objective, therefore, was to evaluate the longitudinal association of circulating levels of n-3 PUFAs with sCD163 and adiponectin, which are established biomarkers of AT inflammation and function, using data from a well-characterized longitudinal observational cohort of adults at high risk for type 2 diabetes (T2D) (24, 25). We also assessed associations with biomarkers of systemic inflammation, including TNF-α, YKL40, C-reactive protein (CRP), and IL-6. We hypothesized that n-3 PUFAs including ALA, EPA, n-3 DPA, and DHA would be inversely associated with AT inflammation.

## Methods

### Prospective Metabolism and Islet Cell Evaluation (PROMISE) Cohort

The present study used data from the ongoing PROMISE cohort, a longitudinal observational cohort study of 736 adults, aged 30 years and older, at high risk for T2D and associated health consequences. This study recruited participants from Toronto and London, Ontario, with baseline data obtained between 2004 and 2006 (24). Clinic examinations were conducted every 3 years. At each clinic visit, participants underwent extensive metabolic characterization and anthropometric measurements and completed standardized lifestyle questionnaires (26-28). The current analysis used data from the baseline examination as well as 2 follow-up assessments over 6 years. After excluding participants without complete measures of the inflammatory biomarkers and n-3 PUFAs and excluding those who did not participate in a follow-up visit, a total of 474 participants were included in this analysis (Figure S1 (29)).

### Outcome Measures: Adipose Tissue Inflammation

At baseline and each follow-up visit, fasting blood samples were collected to determine concentrations of insulin, lipids, fatty acids, and biomarkers of inflammation and nutritional status. For the current analysis, the primary outcome variables were concentrations of sCD163 (a biomarker of macrophage activation) and adiponectin, which are established biomarkers of AT inflammation and function (24, 25). Secondary outcomes included YKL-40, TNF-α, IL-6, and CRP, which are biomarkers of systemic subclinical inflammation (30-33). These biomarkers were available from the first 3 clinical examination time points.

Laboratory measurements were performed at the Keenan Research Centre for Biomedical Sciences, St. Michael's Hospital. Adiponectin was measured using the Meso Scale Discovery sandwich immunoassay singleplex kit (24) (Catalog # K151BXC, RRID: AB_2819056) with a sensitivity of .005 ng/mL. sCD163 was measured using the R&D Quantikine ELISA (R&D Systems, Emeryville, CA) (Catalog # DC1630, RRID: AB_3096052), a solid phase sandwich ELISA with a sensitivity of .613 ng/mL (24). CRP was measured using the Siemens Healthcare Diagnostics BN ProSpec (Siemens Healthcare Diagnostics, Mississauga, Ontario, Canada) (Catalog # LKCRP1, RRID: AB_2750938). IL-6 and TNF-α were measured using MSD multiplex kits (Catalog # K15049D, RRID: AB_2801398), which have an average sensitivity of .09 and .13 pg/mL, respectively. YKL-40 was measured using MSD sandwich immunoassay kit (Catalog # K151VLK-1, RRID: AB_3096053) with a sensitivity of .22 pg/mL. All samples were run in duplicate following the manufacturer's protocol.

### Exposure Measures

Fatty acid measures were conducted using stored serum samples from baseline. Samples were aliquoted and immediately frozen at −80 °C (34) and were not exposed to any freeze-thaw cycles. A modified Folch method (35) including a known amount of internal standard was used to extract the total lipids. A portion of the total lipid extracts were then applied to thin-layer chromatography plates that were developed to isolate the 4 serum lipid fractions: CE, PL, TG, and NE fatty acids (34). These 4 fractions were then converted to fatty acid methyl esters that were then separated and quantified using a Varian-430 gas chromatograph-flame ionization detector. Peaks were identified by retention times of fatty acid methyl ester reference standards. Four n-3 PUFAs were assessed in the PROMISE samples: 18:3 ALA, 20:5 EPA, 22:5 DPA, and 22:6 DHA, which were expressed for the current analysis as mol% and nmol of total fatty acids (34).

### Covariates

Anthropometric measurements were taken using standardized procedures and included waist circumference, weight and height. Body mass index (BMI) was calculated as weight in kg/height in m^2^. Demographics and familial history of diabetes were also assessed at each clinic visit using standardized questionnaires. The Modifiable Activity Questionnaire was used to assess the subject's physical activity; this instrument collects information on the frequency and duration of occupational and leisure activity over the past year (28).

### Statistical Analysis

Continuous variables were described as either mean ± SD for normally distributed variables or median with interquartile range for nonnormally distributed variables, with differences across categories tested using ANOVA. Categorical variables were presented as a number and a percentage; differences were assessed using chi-square testing. Associations between n-3 PUFAs and inflammatory biomarkers at baseline were estimated using Spearman correlations.

The associations of n-3 PUFAs with changes in inflammatory biomarkers were examined using data from the baseline and 3- and 6-year examinations. Statistical analysis was conducted using Generalized Estimating Equation (GEE) analysis to take advantage of the longitudinal nature of the repeated measurements and to account for missing data. Because the fatty acids were only measured at baseline, the n-3 PUFAs were held constant over the 2 follow-up visits. By doing so, the association between baseline serum (mol%) n-3 PUFA (ALA, EPA, n-3 DPA, DHA) with longitudinal change in inflammatory biomarkers was assessed. The outcome variables were log transformed to account for skewness in their distribution. The estimates (beta-coefficients) were thus interpreted as an expected percent difference in the outcome variable (inflammatory biomarkers) for every SD increase in the exposure variable (n-3 PUFA). The GEE models were adjusted for covariates using 2 different models. Covariate model 1 included follow-up visit, baseline age, sex, ethnicity, family history of diabetes, and physical activity. Covariate model 2 included variables in model 1 with the addition of waist circumference. The covariates age, sex, and family history of diabetes were classified as time independent because they were measured at baseline only or did not change with each follow-up visit. *P* values were also adjusted using the Benjamini-Hochberg false discovery rate (FDR) (36) to account for multiple testing.

Interaction tests for sex were conducted to identify possible effect modification in the associations between circulating n-3 PUFAs and circulating inflammatory biomarkers. The significance level was set at *P* < .01 for the identification of any interactions. Subgroup analyses were conducted in the event that any interactions were significant.

All analyses were conducted in R version 4.2.1 and GEE analyses were performed using the R geepack package (https://cran.rproject.org/web/packages/-geepack/index.html).

## Results

Table 1 describes the characteristics of PROMISE cohort participants, overall and separately by sex. At baseline, circulating ALA, EPA, DPA, and DHA represented .69%, .86%, .35%, and 1.32% of total fatty acids, respectively. The mean dietary daily intakes of ALA, EPA, DPA, and DHA were 1.21, .03, .01, and .07 g/day, respectively. Most of the participants were female (n = 348, 73.4%) and 50.8% of participants had a BMI ≥30 kg/m^2^ (mean BMI = 31.1 kg/m^2^).

**Table 1.** Baseline characteristics of PROMISE participants

Measure	Total	Female	Male
N	474	348	126
Age, y	50.0 (9.83)	50.0 (9.54)	50.0 (10.6)
Ethnicity (European, n (%))	333 (70.3)	254 (73.0)	79 (62.7)
MET (kcal/kg/week)	20.1 [7.92, 53.6]	19.3 [7.47, 49.5]	25.9 [9.12, 67.5]
BMI (kg/m^2^)	31.1 (6.43)	31.5 (6.74)	29.8 (5.33)
BMI categories (n (%))			
<25 kg/m^2^	65 (13.7)	51 (14.7)	14 (11.1)
25-29.9 kg/m^2^	158 (33.3)	98 (28.2)	60 (47.6)
≥30 kg/m^2^	241 (50.8)	190 (54.6)	51 (40.5)
Waist circumference (cm)	98.3 (15.4)	96.2 (15.5)	104 (13.7)
Adiponectin (mg/L)	14.3 [10.8, 21.4]	16.0 [11.8, 23.0]	11.9 [8.76, 16.5]
sCD163 (ng/mL)	912 [719, 1041]	935 [719, 1051]	872 [718, 1022]
YKL-40 (mg/L)	31.1 [21.9, 44.4]	31.3 [22.2, 44.1]	30.4 [21.2, 45.3]
TNF-α (pg/mL)	1.89 [1.43, 3.02]	1.87 [1.39, 3.07]	1.98 [1.50, 2.96]
IL-6 (pg/mL)	.87 [.58, 1.33]	.92 [.61, 1.40]	.72 [.54, 1.21]
CRP (mg/mL)	2.00 [1.10, 4.90]	2.60 [1.20, 5.93]	1.50 [.80, 2.28]
Total cholesterol (mmol/L)	5.16 (.90)	5.25 (.90)	4.91 (.86)
HDL (mmol/L)	1.37 (.38)	1.45 (.38)	1.15 (.30)
LDL (mmol/L)	3.12 (.79)	3.15 (.81)	3.04 (.72)
TAG (mmol/L)	1.48 (.77)	1.42 (.70)	1.64 (.92)
Circulating n-3 PUFAs total fatty acids (mol%)			
18:3 (ALA)	.69 (.24)	.69 (.24)	.70 (.25)
20:5 (EPA)	.86 (.51)	.87 (.51)	.85 (.53)
22:5 (DPA)	.35 (.12)	.35 (.13)	.35 (.12)
22:6 (DHA)	1.32 (.53)	1.32 (.54)	1.33 (.52)
Dietary n-3 PUFAs (g/day)			
18:3 (ALA)	1.21 [.84, 1.69]	1.22 [.80, 1.67]	1.19 [.95, 1.74]
20:5 (EPA)	.03 [.01, .06]	.03 [.01, .05]	.03 [.01, .07]
22:5 (DPA)	.01 [.01, .02]	.01 [.01, .02]	.01 [.01, .02]
22:6 (DHA)	.07 [.04, .12]	.07 [.04, .12]	.07 [.04, .13]

Measures are presented as either mean (SD) or median [interquartile range]. Sample sizes vary slightly due to occasional missing values.

Abbreviations: BMI, body mass index; CRP, C-reactive protein; HDL, high-density lipoprotein; LDL, low-density lipoprotein; MET, metabolic equivalent task; sCD163, soluble CD163; TAG, triacylglycerol; YKL-40, Chitinase-3-like protein 1.

Waist circumference declined across increasing tertiles of circulating n-3 DHA (*P* < .05). Differences across tertiles of circulating marine-derived n-3 PUFAs (EPA, DPA, DHA) were also noted for adiponectin (EPA, *P* < .001; DPA and DHA, both *P* < .01), where higher concentrations of adiponectin were observed in the highest n-3 PUFA tertile. Conversely, there were significant differences in sCD163 across DPA tertiles (*P* < .001), with the lowest DPA tertile showing highest concentrations of sCD163 (Supplementary Data Table S1-4 (29)).

**Figure 1.**
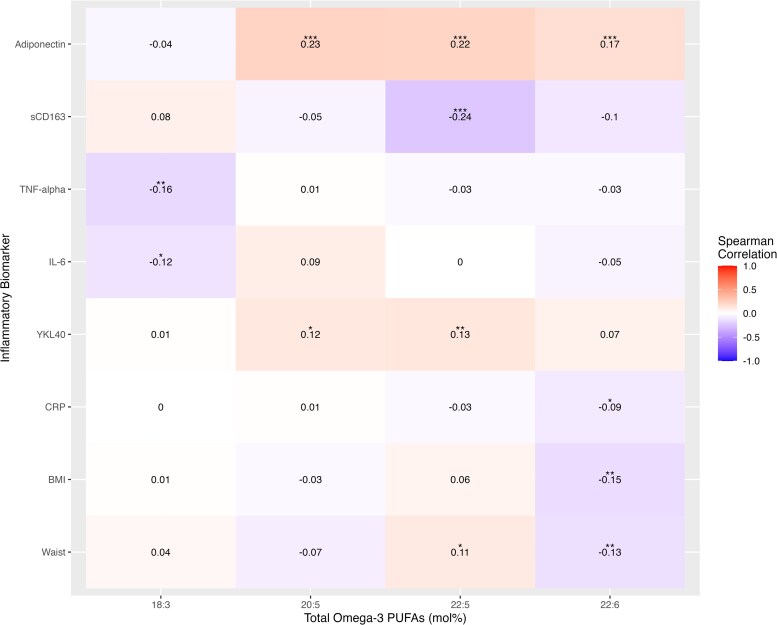
Spearman correlation heatmap of baseline total n-3 PUFAs with inflammatory biomarkers (n = 474). *P < .05, **P < .01, and ***P < .001.

Spearman correlations of total circulating n-3 PUFAs with circulating inflammatory biomarkers and anthropometric measures are shown in Fig. 1. Positive correlations between EPA, DPA, and DHA with adiponectin (*r* = .23, .22, .17, respectively; all *P* < .001) were observed. sCD163 also presented a significant inverse relationship with DPA (*r* = −.24, *P* < .001). Furthermore, significant positive correlations were also observed between EPA and DPA with YKL-40 (*r* = .12 and .13, *P* < .05 and .01, respectively). In contrast, TNF-α and IL-6 showed no significant relationship with the marine-derived n-3 PUFAs, although significant inverse relationships with ALA were noted (*r* = −.16 and −.12, both *P* < .05). CRP was inversely associated with DHA (*r =* −.09, *P* < .05). DHA showed strong inverse associations with both BMI and waist circumference (*P* < .01). In contrast, DPA was positively associated with waist circumference (*r* = .11, *P* < .05).

**Figure 2.**
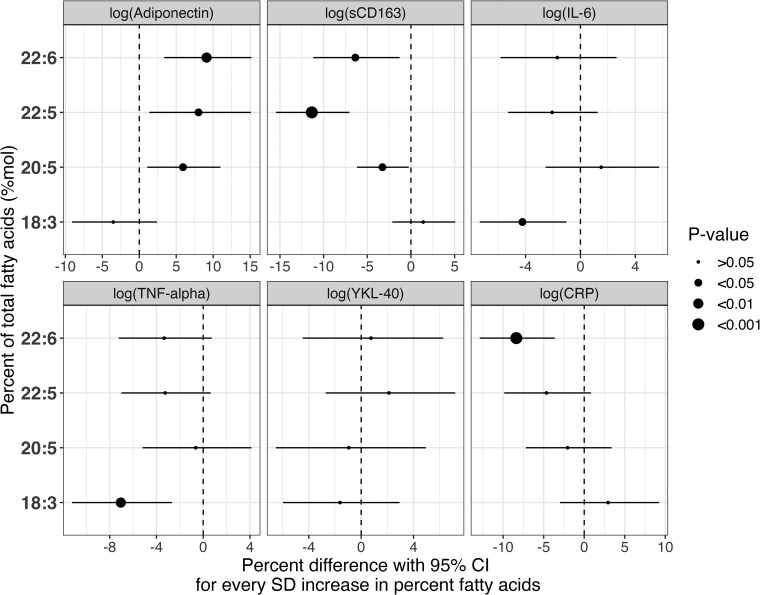
GEE models showing associations between baseline measures of n-3 PUFAs (mol%) and inflammatory biomarkers in the PROMISE cohort over the 6-year follow-up period. This model displays the associations between baseline measures of total n-3 PUFAs and inflammatory biomarkers adjusted using covariate model 1, which includes follow-up, smoking, baseline age, sex, familial history of diabetes, and physical activity (metabolic equivalent task [MET]).

Figure 2 presents associations of total serum n-3 PUFAs with changes in circulating inflammatory biomarkers over 6 years from GEE models. Significant inverse associations were observed between sCD163 and marine-derived PUFAs (DPA: β = −11.4%, 95% CI [−15.5, −7.04], *P* < .001, EPA: β = −3.26%, 95% CI [−6.18, −.253], DHA: β = −6.37%, 95% CI [−11.2, −1.29], both *P* < .05) after adjustment for follow-up, smoking, baseline age, sex, familial history of diabetes, and physical activity. Similarly, the marine-derived n-3 PUFAs had significant positive associations with adiponectin (EPA: β = 5.91%, 95% CI [1.08-11.0], DPA: β = 8.01%, 95% CI [1.35-15.1], both *P* < .05, DHA: β = 9.11%, 95% CI [3.36-15.2], *P* < .01). A significant inverse association was also observed between DHA and CRP (β = −8.37%, 95% CI [−12.9, −3.63], *P* < .001). For the inflammatory biomarkers TNF-α and IL-6, significant inverse relationships were shown with ALA (TNF-α: β = −7.05%, 95% CI [−11.2, −2.68], *P* < .01, IL-6: β = −4.23%, 95% CI [−7.34, −1.02], *P* < .05). In contrast, no significant associations were shown between YKL-40 and the n-3 PUFAs over the 6-year follow-up period.

**Figure 3.**
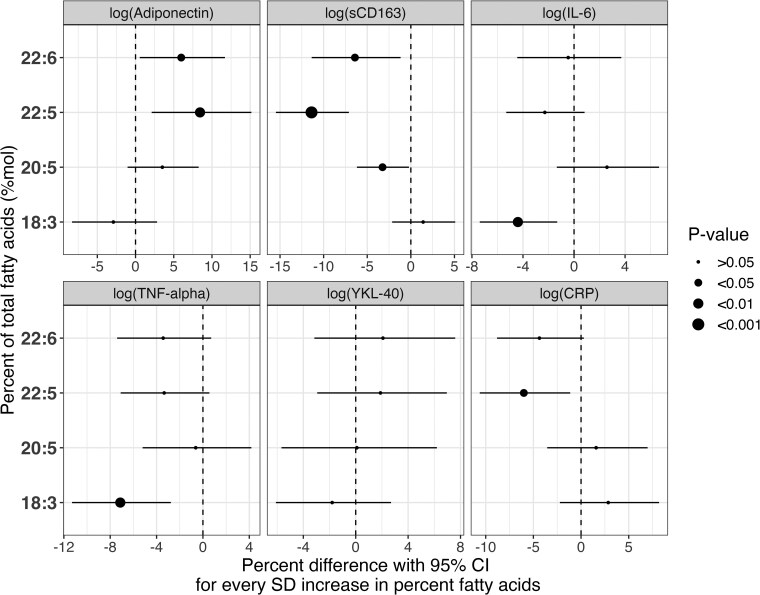
GEE models showing associations between baseline measures of n-3 PUFAs (mol%) and inflammatory biomarkers in the PROMISE cohort over the 6-year follow-up period. This model displays the associations between baseline measures of total n-3 PUFAs and inflammatory biomarkers adjusted using covariate model 2, which includes follow-up, baseline age, sex, familial history of diabetes, physical activity (metabolic equivalent task [MET]), and waist circumference.

Figure 3 presents the GEE models further adjusting for waist circumference (covariate model 2). With this additional adjustment, it was shown that the relationships between adiponectin with EPA and DHA were attenuated. Similarly, the relationship between CRP and DHA was also attenuated with waist circumference adjustment. In contrast, the magnitude of the associations between TNF-α and CRP with DPA increased and were statistically significant with the additional adjustment (TNF-α: β = −4.38%, 95% CI [−7.72, −.939], *P* < .05, CRP: β = −4.86%, 95% CI [−9.44, −.048], *P* < .05). The associations found between sCD163 and marine-derived n-3 PUFAs did not change markedly with the additional covariate adjustment. After FDR adjustment, many significant associations were attenuated; however, significant in the associations between DPA with sCD163 and adiponectin remained (Supplementary Data Figure S2 (29)).

Interaction testing by sex indicated that the association between sCD163 and DPA differed for males and females (*P <* .001), thus a subgroup analysis was conducted. In females, the association was significant (β = −15.4%, 95% CI [−20.3, −10.1], *P* < .001), whereas the association in males was weaker in magnitude and not statistically significant (β = −.441%, 95% CI [−5.42, 4.80], *P > .05)* (Supplementary Data Table S5 (29)).

**Figure 4.**
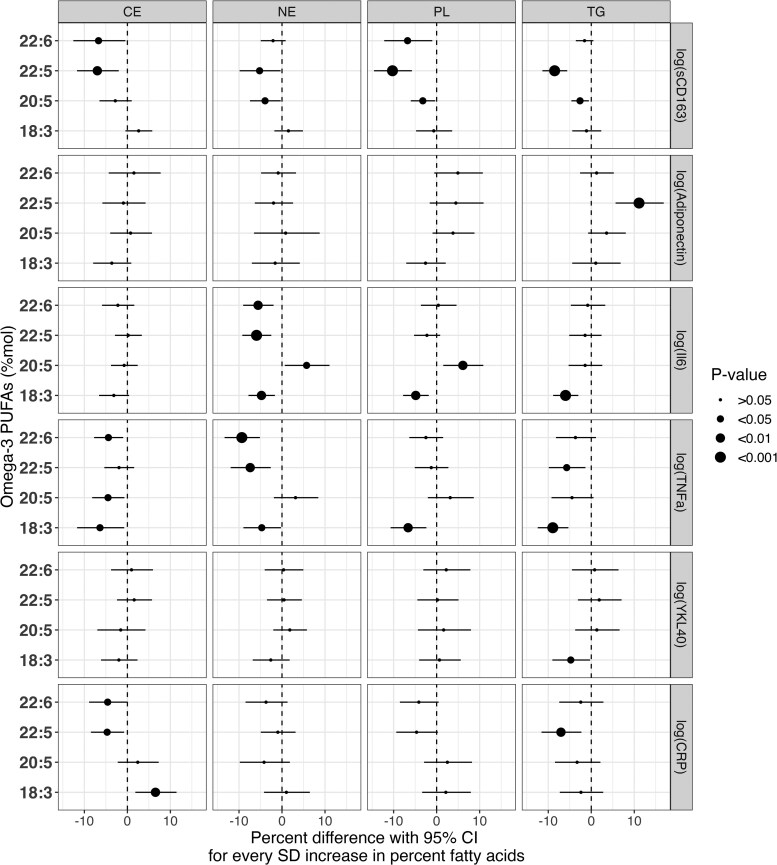
GEE models showing associations between baseline measures of n-3 PUFAs (mol%) and inflammatory biomarkers in the PROMISE cohort over 6-year follow-up time. This model displays the associations between baseline measures of all four serum fractions (cholesterol ester [CE], nonesterified [NE], phospholipid [PL], and triacylglycerol [TG]) of n-3 PUFAs and inflammatory biomarkers adjusted using covariate model 2, which includes, follow-up, baseline age, sex, familial history of diabetes, physical activity (metabolic equivalent task [MET]), and waist circumference.

Figure 4 presents GEE results for associations of n-3 PUFAs in the 4 serum lipid fractions with longitudinal changes in the inflammatory biomarkers over the 6-year follow-up period. Overall, results within the 4 fractions were similar in magnitude and direction to the associations observed in the total lipid pool. After adjustment for covariates using model 2, a number of significant inverse associations were observed across all 4 fractions for sCD163, especially with the marine-derived n-3 PUFAs. Similarly, several significant inverse associations were observed across all 4 pools with inflammatory biomarkers sCD163, IL-6, and TNF-α. Notably, adiponectin had no significant associations except in the TG pool. IL-6 only showed significant associations with ALA in the total lipid pool, however, was significantly associated with all n-3 PUFAs in the NE pools. Similarly, more significant associations were seen between n-3 PUFAs and TNF-α in both the CE and NE pools in comparison to the total lipid pool. After FDR adjustment, many significant associations across all 4 pools were attenuated; however, significant associations remained in NE and TG pools across the marine-derived n-3 PUFAs for sCD163, adiponectin, IL-6, and TNF-α (Supplementary Data Figure S3 (29)).

## Discussion

This 6-year longitudinal observational study examined the association of n-3 PUFAs with measures of AT inflammation. We showed that in a Canadian population of adults at risk for T2D, those with higher serum proportions of marine-derived n-3 PUFAs within the total lipid pool at baseline had significantly lower levels of sCD163 in the following years. Furthermore, a significant positive association was observed between baseline DPA and DHA in the total lipid pool with longitudinal measures of adiponectin over the 6-year follow-up period. Generally consistent associations were observed between circulating n-3 PUFAs and both adiponectin and sCD163 across all 4 serum lipid fractions. The observed relationships align with our initial hypotheses, namely that baseline serum levels of n-3 PUFAs have an inverse association with longitudinal markers of AT inflammation.

Studies in animal models have supported a link between n-3 PUFAs and reduced AT inflammation (16, 18, 37); however, fewer data are available from human studies. Albracht-Schulte et al conducted a comprehensive review summarizing the role of n-3 PUFAs in obesity and metabolic syndrome in both animal models and human studies; the review concluded that the antiobesity of effects of n-3 PUFAs were shown in animal models in contrast to inconclusive results in human studies, specifically regarding the relationship between n-3 PUFA supplementation and body weight (20). Other animal model studies using direct AT biopsies found that n-3 PUFA supplementation significantly reduced inflammatory processes at the cellular and gene expression level (16, 18, 19, 37). Previous human RCTs examining the effect of n-3 PUFA supplementation on general inflammatory markers, such as CRP and TNF-α, have shown heterogeneous results (17, 18, 23, 30, 31, 33, 38, 39). Fewer studies have focused specifically on AT inflammation in humans using targeted AT biomarkers and/or biopsies (22, 32, 40-43). Many of these studies have been smaller, short-term RCTs focusing on oral supplementation of n-3 PUFAs using fish oil (22, 40-43). An RCT involving adults with elevated CRP levels found a significant reduction in CRP levels following n-3 PUFA supplementation with EPA and DHA capsules (38). Similarly, another recent RCT found that individuals supplemented with EPA and DHA had a significant reduction in inflammatory markers such as IL-6 and TNF-α (30). A recent meta-analysis of 7 RCTs showed that n-3 PUFA supplementation significantly increased serum adiponectin in adults with T2D (21). Contrary to these findings, other RCTs have concluded that n-3 PUFAs did not significantly impact AT inflammation (22, 33, 44). To our knowledge, no large longitudinal observational study has previously been conducted examining the possible link between n-3 PUFAs and AT inflammation using adipose specific inflammatory biomarkers.

In the current study, a significant difference between sexes was observed in the associations between sCD163 and DPA. Specifically, the association was significant among females but not in males. Although this finding may be due to the smaller sample of male participants in this study (resulting in lower statistical power), the observation is consistent with current literature. It has been consistently demonstrated that females have higher circulating EPA, n-3 DPA, and DHA levels in comparison to men because of differing elongation conversion rates within the body (45, 46); thus, the weaker association in males may be due to lower levels of circulating DPA in men in comparison to women (45).

Our study extends the current literature on the relationship between n-3 PUFAs and AT inflammation in humans. We assessed the association of baseline serum n-3 PUFAs with longitudinal measures of established biomarkers of AT inflammation over a 6-year follow-up period. Specifically, we measured adiponectin, an adipokine that is synthesized and secreted by AT that has been documented to be inversely correlated with obesity-associated outcomes including T2D (25). Additionally, we used sCD163, a biomarker of AT macrophage activation (24), allowing us to assess the impact of n-3 PUFAs on the progression of the earliest changes that initiate the AT inflammation cascade (24). We also measured more general biomarkers of inflammation including CRP, TNF-α, IL-6, and YKL-40 to evaluate associations of serum n-3 PUFAs with measures that have been more commonly used in this literature.

The biological mechanism underlying the relationship between n-3 PUFAs and AT inflammation is being actively investigated (9-11, 47, 48). Obesity is characterized by enlarged adipocytes and AT containing high concentrations of macrophages (9). Triggering events that cause inflammation in adipose tissue fall into 3 categories: adipocyte death, hypoxia, and mechanical stress to the adipocyte (9). These triggers may activate the M1 pro-inflammatory macrophage pathway which allows the adipocyte to recruit more M1 macrophages and produce pro-inflammatory cytokines (9). The activation of this pathway leads to an increase in the M1:M2 macrophage ratio, thus shifting the macrophage population towards a pro-inflammatory phenotype. The increased presence of macrophages through recruitment in combination with increased phenotype switching (ie, from M2 to M1) is a hallmark of AT inflammation characterizing obesity and similar chronic inflammatory diseases (11, 14, 47). In healthy individuals, the process by which this AT inflammation is resolved occurs through a range of specialized pro-resolving lipid mediators (SPMs). SPMs are a superfamily of a group of bioactive molecules including resolvins, protectins, and maresins (10). These mediators exert control over the resolution of inflammation and act as potent local resolution agonists by allowing monocytes to differentiate into M2 (anti-inflammatory) macrophages that terminate the inflammatory response (10). The documentation of these pathways has provided evidence that the resolution of inflammation is not a passive process. Notably, it has been shown that n-3 PUFAs, more specially EPA and DHA, are precursors to these SPMs (10, 48). Thus, the inverse relationship of n-3 PUFAs and AT inflammation documented in the literature plausibly occurs at least in part through pro-resolving mediation via SPMs.

This study has several strengths. First, a well-characterized longitudinal cohort of subjects at risk for T2D with baseline and 2 follow-up visits was used. Extensive covariate data included demographic, anthropometric, and metabolic measures at each time point. Second, serum n-3 PUFAs were measured in 4 serum lipid pools, which allowed us to compare the associations of n-3 PUFAs from different lipid fractions. Third, inflammatory biomarker concentrations were measured at multiple time points allowing for detailed longitudinal analyses of their associations with the n-3 PUFAs. Finally, this study used GEE models to take advantage of the longitudinal repeated measurements and allowed for the maximum number of participants to be used in the analyses.

There are also some important limitations to consider when interpreting the results of this study. First, PROMISE is an observational cohort and therefore residual confounding may be present. Also, because of the observational nature of this study, causal associations between n-3 PUFAs and inflammatory biomarkers could not be made. Second, it is recognized the adipose tissue biopsies are the gold standard for characterizing AT inflammation; however, this study was limited to using circulating biomarkers because of the unavailability in the PROMISE cohort of more direct measures such as AT biopsies. Another limitation was that n-3 PUFAs were only measured at 1 timepoint. Further, the PROMISE cohort mainly comprised female participants of European descent at risk for T2D; therefore, these results may not be generalizable to populations not of the same demographic. Finally, because we did not have access to AT biopsies, our characterization of AT inflammation relied on the use of biomarkers. Nonetheless, both sCD163 and adiponectin are established measures of AT inflammation/function.

## Conclusion

Our findings suggest that those with higher serum n-3 PUFAs at baseline have a significantly altered levels of AT inflammatory biomarkers (higher sCD163 and lower adiponectin) in the following years in a Canadian population at risk for T2D. These results will add to the limited evidence on the potential role of n-3 PUFAs in the prevention and management of AT inflammation in humans. Future research is needed using direct measures of AT inflammation from biopsies to further our understanding on the role of n-3 PUFAs in AT inflammation. These findings may help form interventions specifically targeting AT inflammation, which is an emerging risk factor for chronic diseases such as obesity, T2D, and cardiovascular diseases.

## Data Availability

Some or all datasets generated during and/or analyzed during the current study are not publicly available but are available from the corresponding author on reasonable request.

## Abbreviations

ALA: alpha-linolenic acid
AT: adipose tissue
BMI: body mass index
CE: cholesteryl ester
CRP: C-reactive protein
DHA: docosahexaenoic acid
DPA: docosapentaenoic acid
EPA: eicosapentaenoic acid
FDR: false discovery rate
GEE: Generalized Estimating Equation
n-3: omega-3
NE: nonesterified fatty acids
PL: phospholipid
PUFA: polyunsaturated fatty acid
RCT: randomized controlled trial
SPM: specialized pro-resolving lipid mediator
T2D: type 2 diabetes
TG: triacylglycerol
